# Male sterility in Mcl-1-flox mice is not due to enhanced Mcl1 protein stability

**DOI:** 10.1038/cddis.2016.391

**Published:** 2016-12-01

**Authors:** Casey Ah-Cann, Maximilien Tailler, Andrew J Kueh, Marco J Herold, Joseph T Opferman, Marie-Liesse Asselin-Labat, Philippe Bouillet

**Affiliations:** 1 Molecular Genetics of Cancer, The Walter and Eliza Hall Institute of Medical Research, Parkville, Victoria 3052, Australia; 2 Department of Medical Biology, The University of Melbourne, Melbourne, Victoria 3052, Australia; 3 Department of Cell and Molecular Biology, St. Jude Children's Research Hospital, Memphis, TN, USA

*Dear Editor*,

The myeloid cell leukemia sequence 1 (*Mcl1*) gene is essential for development and tissue homeostasis throughout life. Homozygous deletion of *Mcl1* alleles leads to the death of the embryos at the time of implantation. We have previously engineered a conditional (Mcl1-flox) allele of the gene and reported that the insertion of a loxP site in the first exon had inadvertently led to the addition of 13 amino acids at the N-terminus of the coding sequence.^1^ The Mcl1 protein is turned over rapidly, and several mechanisms have been implicated in the regulation of Mcl-1 levels.^2, 3, 4^ Interestingly, the extended Mcl1 protein (termed N^+^Mcl1) encoded by the Mcl1-flox allele appeared more stable than the wild-type protein. Significantly, homozygous Mcl1^fl/fl^ males were sterile, showing impaired sperm development and complete absence of mature sperm in the epididymis. This phenotype was indistinguishable from that observed in mice lacking both Bim and Blk.^5^ Since proper testis development and sperm maturation appear to require a fine balance between cell death and cell survival, we surmised that a more stable Mcl1 protein in Mcl1^fl/fl^ males somehow altered this balance and cause the testicular phenotype.

Since a more stable Mcl-1 protein may influence the outcome of some experiments in unpredictable ways, we have now used the CRISPR/Cas9 technology to remove the sequence encoding the 13 extra amino acids from the Mcl1-flox allele, giving rise to the Mcl1-flox-del strain. As shown in Figure 1a, and confirmed by sequencing of the locus, the Mcl1 protein produced from the Mcl1-flox-del allele had the same size as the WT protein, while the addition of 13 amino acids in the N^+^Mcl1 protein resulted in a higher molecular weight protein as observed by western blot. Even though the Mcl-1 protein in Mcl1-flox-del homozygous animals is normal, the males were still infertile. As observed in the Mcl1-flox strain, seminiferous tubules of homozygous Mcl1-flox-del males contained some round spermatids as well as spermatocytes, but lacked 1N spermatids and had essentially no mature sperm. Accordingly, their epididymides were devoid of sperm, like those of homozygous Mcl1-flox mice (Figure 1b). These results thus exclude that the enhanced stability of the N^+^Mcl1 protein is the cause of the testicular phenotype. Interestingly, an independent conditional Mcl1 KO strain has been generated, in which the first loxP site was introduced upstream of the first exon, and the coding sequence unmodified.^6^ Homozygous males of this strain were also infertile, confirming our present result.

Although our results fail to provide an explanation for the impairment of spermatogenesis in mice carrying a floxed *Mcl1* allele, it seems reasonable to assume that the introduction of a loxP site close to *Mcl1* first exon may have unintended effects on the expression of the gene, at least in the testis. Surprisingly, several studies have shown that the floxed alleles do not provide any survival advantage to the many hematopoietic cells studied to date in *in vitro* death assays,^6, 7, 8^ suggesting that this effect may be specific to the testis. More work remains to be done to fully understand the intricacies governing *Mcl1* expression in different tissues.

## Figures and Tables

**Figure 1:**
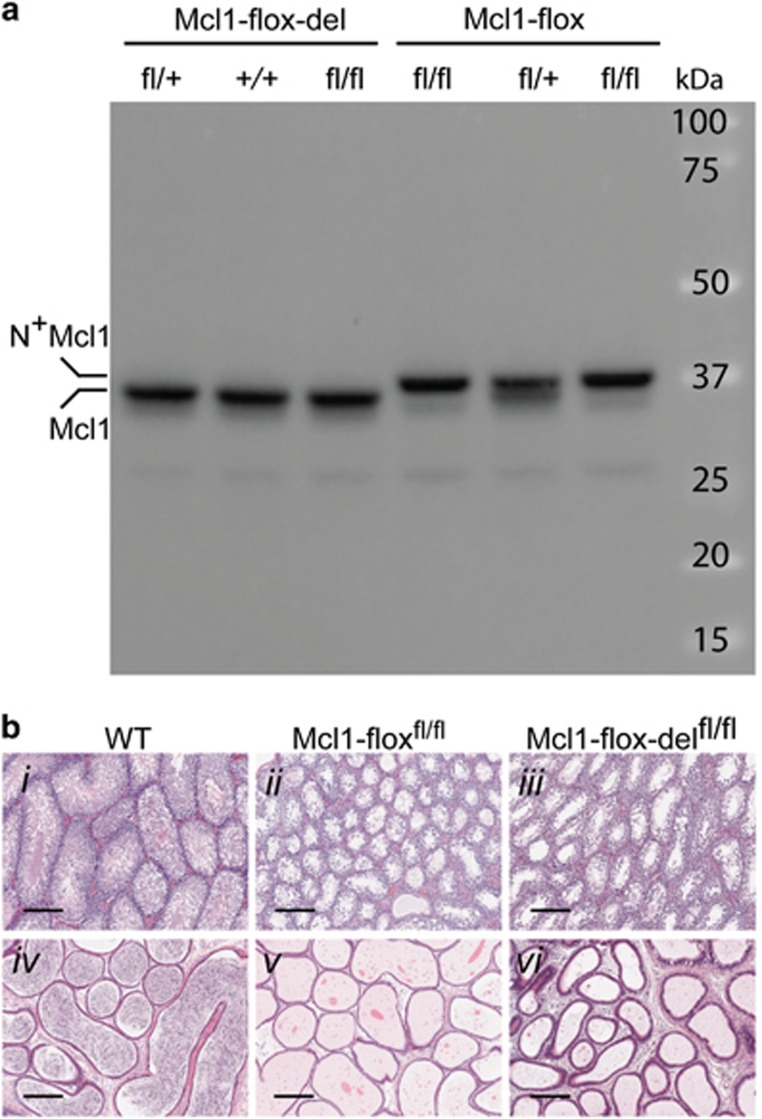
Spermatogenesis defect in Mcl1-flox-del mice. (**a**) Detection of Mcl1 protein in thymic extracts from Mcl1-flox-del and Mcl1-flox mice. Note the size correction in Mcl1-flox-del mice compared to the protein from Mcl1-flox mice. (**b**) Sections through testis (i−iii) and epididymis (iv−vi) from WT, Mcl1-flox and Mcl1-flox-del homozygous mice. Note the complete absence of mature sperm in the epididymides of both floxed strains
